# Development of mHealth system for supporting self-management and remote consultation of skincare

**DOI:** 10.1186/s12911-015-0237-4

**Published:** 2015-12-30

**Authors:** Bambang Parmanto, Gede Pramana, Daihua X. Yu, Andrea D. Fairman, Brad E. Dicianno

**Affiliations:** Department of Health Information Management, University of Pittsburgh, 6026 Forbes Tower, Pittsburgh, PA 15260 USA; Department of Occupational Therapy, MGH Institute of Health Professions, Boston, MA 02129 USA; Department of Physical Medicine and Rehabilitation, University of Pittsburgh, Pittsburgh, PA 15260 USA

**Keywords:** Mobile health (mHealth), teledermatology, telehealth, telemedicine, spina bifida, self-management, telecare

## Abstract

**Background:**

Individuals with spina bifida (SB) are vulnerable to chronic skin complications such as wounds on the buttocks and lower extremities. Most of these complications can be prevented with adherence to self-care routines. We have developed a mobile health (mHealth) system for supporting self-care and management of skin problems called SkinCare as part of an mHealth suite called iMHere (interactive Mobile Health and Rehabilitation). The objective of this research is to develop an innovative mHealth system to support self-skincare tasks, skin condition monitoring, adherence to self-care regimens, skincare consultation, and secure two-way communications between patients and clinicians.

**Methods:**

In order to support self-skincare tasks, the SkinCare app requires three main functions: (1) self-care task schedule and reminders, (2) skin condition monitoring and communications that include imaging, information about the skin problem, and consultation with clinician, and (3) secure two-way messaging between the patient and clinician (wellness coordinator). The SkinCare system we have developed consists of the SkinCare app, a clinician portal, and a two-way communication protocol connecting the two. The SkinCare system is one component of a more comprehensive system to support a wellness program for individuals with SB.

**Results:**

The SkinCare app has several features that include reminders to perform daily skin checks as well as the ability to report skin breakdown and injury, which uses a combination of skin images and descriptions. The SkinCare app provides reminders to visually inspect one’s skin as a preventative measure, often termed a “skin check.” The data is sent to the portal where clinicians can monitor patients’ conditions. Using the two-way communication, clinicians can receive pictures of the skin conditions, track progress in healing over time, and provide instructions for how to best care for the wound.

**Conclusions:**

The system was capable of supporting self-care and adherence to regimen, monitoring adherence, and supporting clinician engagement with patients, as well as testing its feasibility in a long-term implementation. The study shows the feasibility of a long-term implementation of skincare mHealth systems to support self-care and two-way interactions between patients and clinicians.

## Background

Individuals with spina bifida (SB) are susceptible to hospitalization due to problems related to sepsis, urinary tract infections (UTIs) and skin wounds [1, 2]. Skin problems (chronic ulcers, infections and other wounds) are among the top three primary diagnoses for hospitalization [1]. Nearly one in three of patients with SB had at least one wound episode in a recent open-cohort study [3]. People with SB have to be vigilant in checking for skin injury and breakdown. Pressure ulcers are the most common cause of skin injury resulting from prolonged sitting or braces. Loss of sensation in the lower body associated with spinal cord lesions means there is no trigger to indicate a need to reposition oneself and reduce the pressure on a particular part of the body. Incontinence causes the skin to become even more susceptible to breakdown and infection. Furthermore, the lymphatic and circulatory systems—which act together to remove fluid and waste products—of persons with SB often do not function as efficiently as they should [4]. Without proper functioning of these systems, nutrients and oxygen are not received in adequate supply and edema will accumulate in the lower extremities [5]. These combined issues in persons with SB mean that pressure ulcers not only develop very quickly, but also tend to heal very slowly [6].

The majority of persons with SB have had a wound primarily caused by prolonged pressure [7]. The incidence of pressure ulcers in this group correlated with incontinence and lack of adherence to self-care programs [8–10]. Treatment of pressure ulcers can include dressings, topical medications, debridement and even surgery. Relief from pressure is critical to allow for healing of the affected area. Daily activities to prevent the development of pressure ulcers are necessary components of self-care for persons with SB [11]. These self-care activities include: inspection of the body, especially the buttocks and all sides of the feet and between toes, to detect any changes in the skin, and checking for cuts, bruises, scratches, swelling, and red marks.

The purpose of the SkinCare system is to support a patient’s self-management of skin conditions, to monitor a patient’s adherence to self-care tasks, and to support clinician-directed wound/skin self-care treatments. Lack of adherence to self-care routines and healthcare recommendations can quickly lead to a decline in health status and development of secondary conditions such as pressure ulcers. In addition to reminding the patient to perform regular skin checks and, if necessary, wound care, the SkinCare system provides a line of communication with the clinician (wellness coordinator) to help patients manage complex self-care routines. Therefore, the SkinCare system requires the following components:

- Scheduled reminders for skincare. This function allows patients to schedule their self-care routines and the app provides reminders at the scheduled times. The schedule can also be set up by clinicians remotely.
- Self-care adherence monitoring. The patient’s responses to the self-care schedule synchronize with the portal for a clinician to monitor if the patient is adhering to their self-care routines.
- Skin imaging for monitoring and consultation. This function allows patients to take pictures of wounds and then seamlessly send them to the portal for clinicians to evaluate along with clinically relevant information (size, color, smell, etc.) of the wounds. Using the images and clinically relevant information, the clinician can provide direction on how to care for the wounds.
- Secure two-way messaging between the smartphone app (patient) and clinician portal (wellness coordinator). Using secure messaging, the patient and clinician (using portal or smartphone) can communicate with each other securely to discuss skin problems.

Recent advances in mHealth provide unprecedented opportunities for developing innovative health services and interventions that take advantage of the novel characteristics of mobile technologies [12]. Among those characteristics are interactivity, personalization, and timeliness. In teledermatology practice, a service delivery model that includes interactions between patient and healthcare professional is referred to as patient-enabled teledermatology [13]. This novel service delivery model is made possible by the robust advances in information and communication technologies that allows consistent and reliable communications between patients and clinicians. In the context of individuals with chronic and complex conditions, such as individuals with SB, such higher patient-clinician information engagement can significantly improve the patient's ability to adhere to preventative medical plans [14]. The remainder of the paper is organized as follows: the Methods section describes the SkinCare within the iMHere app suite, the architecture of the SkinCare system, and the setting of the clinical implementation; the Results and Discussion section describes the details of the SkinCare system (including the apps, the portal, and the secure messaging), and the results of the clinical implementation (usage of the apps and the utilizations of the reminder, imaging, and the messaging), and the limitations of the study); followed by Conclusion.

## Methods

We implemented the SkinCare mHealth system as part of a prospective randomized study on the use of an mHealth suite of apps called iMHere (interactive mobile health & rehabilitation) in support of a SB Wellness program [15]. This study was approved by the Institutional Review Board of the University of Pittsburgh, and all participants signed written informed consent. This study is part of a larger study (Mobile Health Self-Management and Support System for Chronic and Complex Health Conditions) that is has been registered in ClinicalTrial.gov with registration number: NCT0259229. Participants were recruited from a local adult SB clinic and local community organizations. Inclusion criteria were: age 18 to 40 years, primary diagnosis of myelomeningocele with hydrocephalus, passing a screening that demonstrated ability to use a smartphone, and living in a community setting within 100 miles of the testing site to allow for technical support. Exclusion criteria were: a diagnosis of severe intellectual disability, a diagnosis of non-myelomeningocele subtype of SB, and severe and persistent psychiatric illness and/or drug or alcohol addiction.

The SkinCare app is the most complex of the iMHere system. In the wellness program, a wellness coordinator (WC) supervised the care of individuals with chronic or complex medical needs. The WC identified the issues that a given patient faced and then designed an individualized plan of care by actively engaging the patient in the process. She encouraged daily skin checks, monitored patient self-care routines, evaluated skin pictures sent by patients, and consulted with a physician for wound treatment. Her role as a liaison and director of care empowered the individuals to be responsible for their own care. A previous pilot study of a wellness program without mHealth support produced improved outcomes with respect to medical complications and health care utilization measures, such as lower rates of skin breakdown (9.7 %) and UTIs (16.1 %) compared to those in the general SB population [16, 17]. As the cost of treating Stage IV wound for community dwelling adults can exceed $124,000 per wound [17], this program’s reduction in the incidence of wounds alone would allow for a significant overall decrease in health care costs.

### Architecture of the SkinCare system

**Fig. 1:**
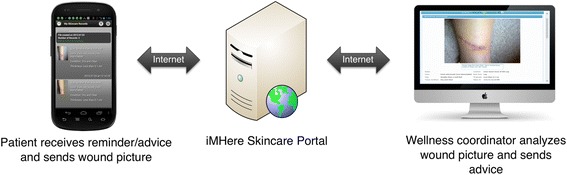
SkinCare System Architecture

The architecture of the SkinCare system consists of a smartphone app, a clinician portal, and a two-way communication connecting them as illustrated in Fig. 1. The app consists of a user interface that interacts with the patient and a background service that maintains a real-time data connection with the portal when the smartphone is in a hibernation mode. This architecture allows the smartphone to alert patients as new data was pushed from the portal (e.g. wound care instructions or secure message sent by a clinician) even when the phone is not active.

The clinician portal is a web-based system designed for clinicians to engage and monitor patients. The primary users of the health portal would be the WC (typically social workers, nurse coordinators, case managers, and/or patient advocates) directly supporting patients. The secondary users of the portal would be physicians, consulted by the WC when interventions required clinical supervision. Clinicians would use desktop, laptop, or tablet computers to access the health portal. Using the portal, the WC monitors a patient’s adherence to the skin self-care routines that have been scheduled in the app, views new skin images, views skin problems, provides instructions on how to care for the skin problem by annotating the image, and exchanges messages with the patients.

Although there is a casual similarity between this architecture and a mobile teledermatology system called “Mobil Neurodermitis” developed by Berndt et al. [18], the underlying functions and capabilities are significantly different. Both architectures utilize a two-part telematic structure that is comprised of a mobile app for the patients and a web-based portal for the clinicians with the mobile app allowing images to be captured and transmitted to the portal. While “Mobil Neurodermitis” is designed as a one-way system for capturing and sending skin pictures from patient (smartphone app) to the clinician portal, the iMHere SkinCare app is designed to provide two-way communication between patient app and clinician portal. The clinician can add directions on how to care for a skin problem, attach it to the skin image, and send it to the patient’s app. In addition, the smartphone app in the SkinCare system is designed to support self-care by patients, with reminders and education, not just image capture.

### Implementation of the system

We implemented the iMHere SkinCare system to support a wellness program for individuals with spina bifida. The SkinCare system was deployed as part of the app suite called iMHere that includes medication management and reminders, catheterization scheduling and reminders, bowel program reminders, and mood tracking [15]. This study was a sub-study of a prospective randomized study conducted for one year with staggered enrollment. The patients were located in a tri-state area (Pennsylvania, Ohio, and West Virginia) within a 100-mile radius of Pittsburgh. Fourteen patients participated in the telehealth group, where more than half of the patients were located in rural areas with the furthest distance being a 2.5 hour drive from Pittsburgh. One patient dropped out because of a very poor cellular phone signal in the area where the patient lived. There was no drop out for reasons other than a poor signal.

## Results and discussions

The SkinCare system consisted of three main components: 1) SkinCheck app for patients, 2) clinician portal and 3) bi-directional secure communication system that connects the app and the portal in real-time. The system has a proactive, preventative component, but also provides support if a skin problem occurs.

### SkinCheck App

The SkinCheck app consisted of four main features: reminders, skin imaging and reporting, wound tracking, and secure messaging. The reminder feature is designed to allow a patient to schedule a daily visual inspection or wound care task. A patient can set up her/his own schedule, choose a time, ringtone or vibrate, and add notes such as the body area that needs to be checked. The schedule can be repeated every day—or any day of the week—and can be modified or removed. The reminder will pop up at the scheduled time, sounding the ringtone associated with it. The reminder will continue to sound until the patient clicks “Check Skin,” confirming that the patient has received the reminder and is proceeding to perform a visual inspection or care for the affected area.

**Fig. 2:**
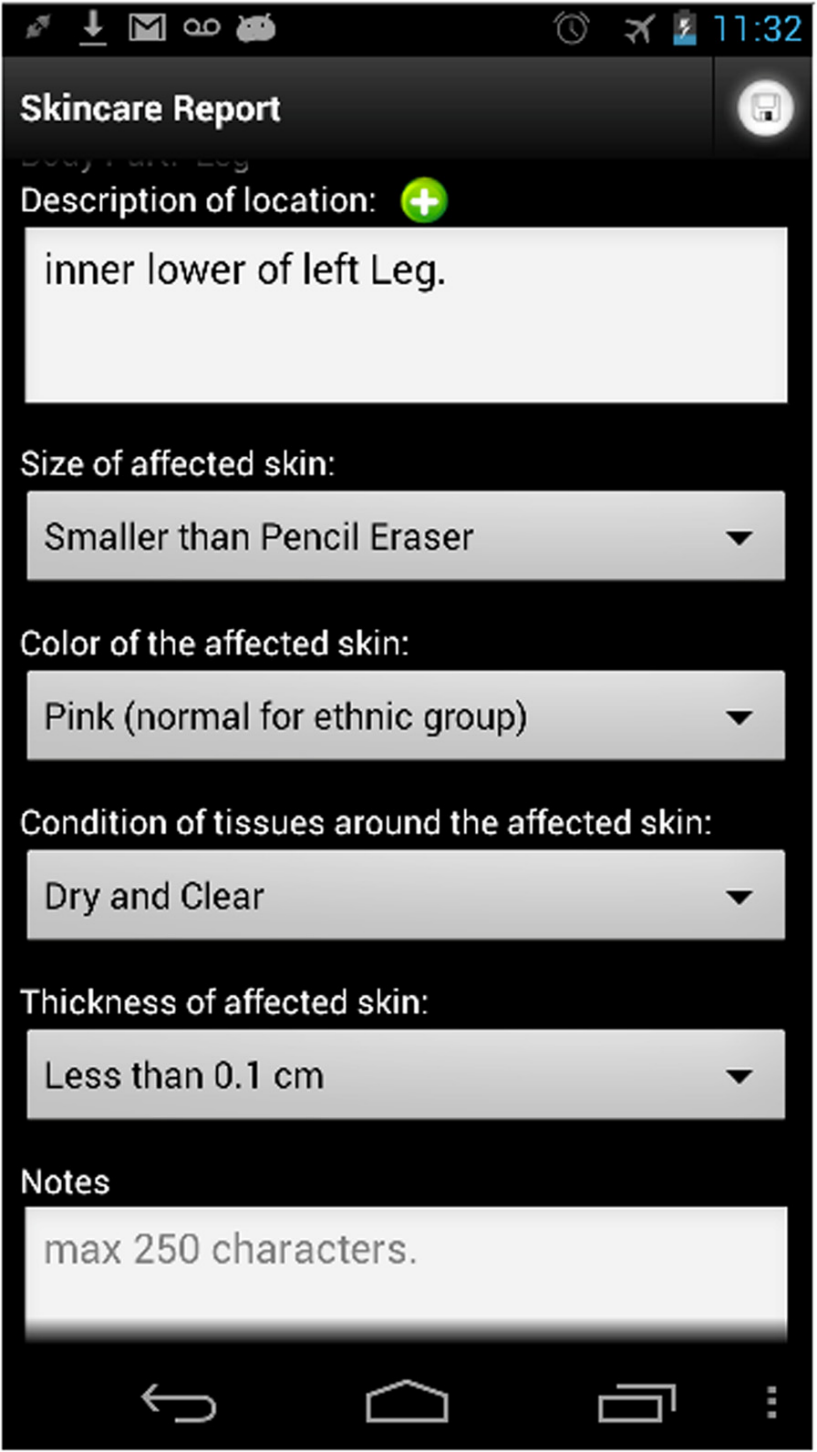
The SkinCheckApp has several screens to allow the user to report wounds

If during the skin check, the patient identifies a new wound, the app will allow the user to report the wound and send the information to the clinician portal. The report consists of imaging and descriptions (See Fig. 2). To help patients create a new report, a color-coded diagram of the human body and a corresponding color-coded zone list are provided to aid in ease of describing the location of the affected area (Fig 2. far left). After the patient selects a body area, the app will bring up the Skincare Camera Viewfinder (Fig 2. middle left) where the patient can use the phone’s camera to take one or more photos of the wound. Patients can provide descriptions of the wound including the location (left, right, upper, or lower), size, color, condition around the affected skin, depth, and general notes (Fig 2. middle right). These descriptions are to help a clinician better understand the condition. This detailed information is also used to flag photos that warrant immediate attention from clinicians. Patients enter the skin report using a dropdown box (pick list) to reduce data entry burden.

The design of the SkinCheck app provides a third feature allowing patients to track the progress of skin problems (Fig 2. far right). Skin images taken periodically for comparison can be organized and tracked for progress. SkinCheck can track as many skin problems as needed, keeping records and photos from each separate skin problem organized together as a single case. The organization of pictures into cases allows the clinician and patient to easily track progress by comparing the pictures over time. The SkinCare app’s main screen shows all cases, with a picture of the most current record from each case. The patient can click a case, and the app will show all of the records for that skincare case, starting with the most recent.

### Clinician portal and messaging

The web-based clinician portal is designed to allow clinicians to monitor a patient’s condition and send a treatment plan to the patient’s smartphone. The portal stores all of the data sent from the SkinCare app including: skin images and reports, a patient’s adherence to reminders, and messages from a patient. The portal is designed as a dashboard, divided into three main areas. On the left is a patient roster and status of the smartphone app (active/connected is indicated by a green check, otherwise it is indicated by a cross). Clinicians can review patients’ self-care schedules, modify, or create new schedules. The primary function of the portal is the SkinCare Review, located in the middle of the portal. The organization of the skin images in the portal is similar to that of the image tracking on the smartphone app. Clinicians can open a case that consists of a series of images by expanding a case folder. Cases can be marked as closed, and this will be indicated to the patient, though it is not possible to delete records from the portal. The series of images provides the opportunity for clinicians to compare the skin conditions over time that enhances the capability for clinicians to diagnose the skin conditions and to detect skin problems sooner. Previous studies [19] have proven that the effectiveness in diagnostics dermatologists relies, among other variables, on constant or repeated examinations. Using this portal, clinician can get access to the repeated pictures without having patients visiting the clinics. The capability of having repeated skin pictures remotely can potentially reduce the cost of healthcare while maintaining good quality of care [20]. The capability of detecting skin problem is one of the potential advantages of the mobile health system [21, 22].

**Fig. 3:**
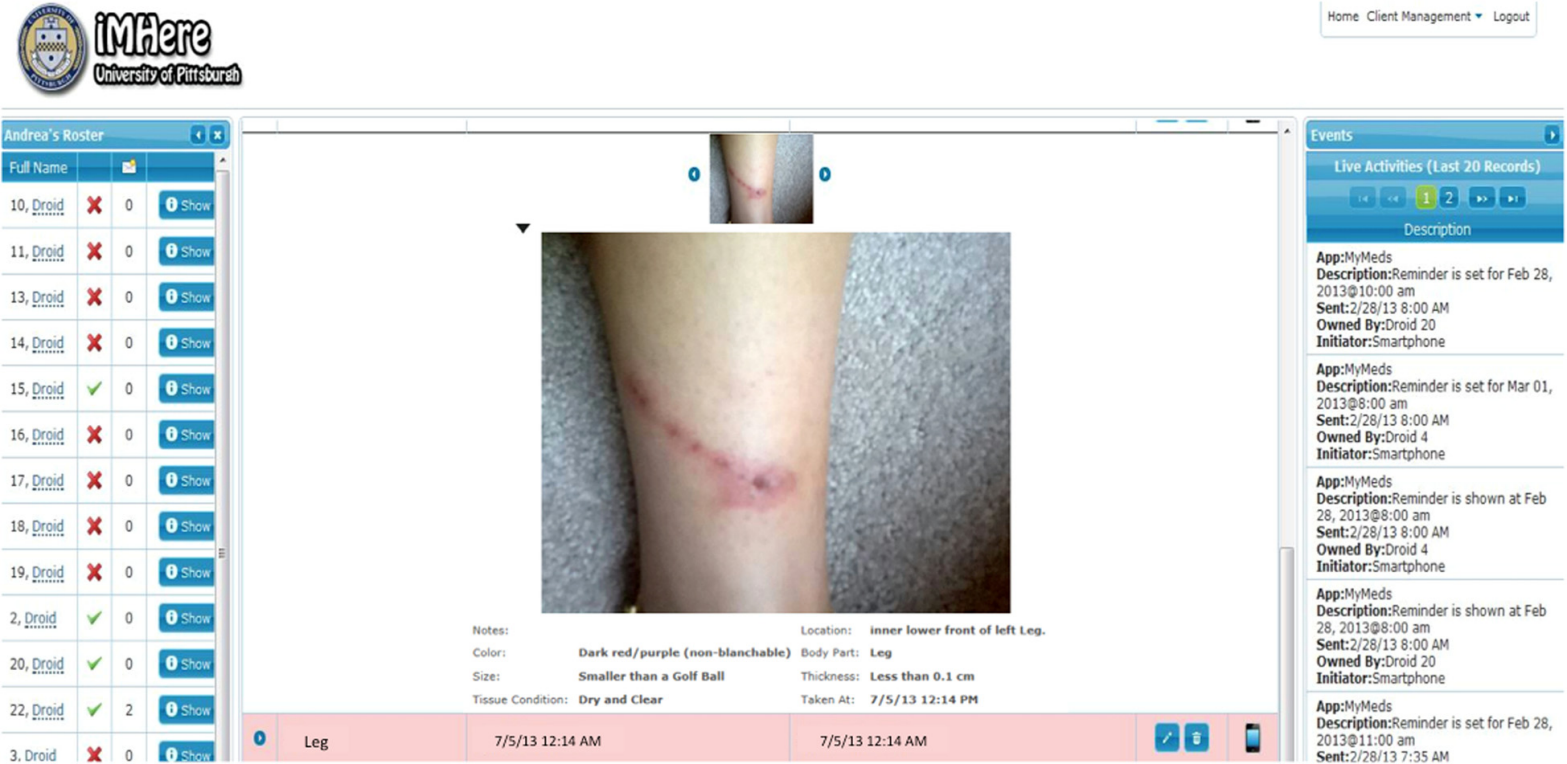
Clinician Portal of the SkinCare system that allows monitoring of wound healing process

Using the portal, clinicians can be actively involved in supporting a patient’s skin self-care. The clinician can add notes containing directions on how to care for a wound or a skin problem to each image the patient takes. These notes are sent to the patient and attached to the corresponding record on their device. The third function of the portal is to allow clinicians to engage patients using the secure messaging feature (Fig. 3 right), which works in a way similar to text messaging. The purpose of the messaging is to allow clinicians and patients to engage in a consultation, discussing skin problems and how to conduct daily self-care routines.

### Usage of the App

The SkinCare system was implemented on an Android smartphone, and the versions of the Android system include versions 2.3 to the latest, 4.1. During the implementation, we did not find serious problems with the fragmentation of the Android operating system other than glitches with the picture-taking function. We found that picture-taking in the SkinCare app did not work in the Android 4.x, despite working well in Android 2.x. After debugging, we found that Android 4.x has faster threads in the operating system, which are not synchronized with the components related to the smartphone camera. The result is that the picture object could not be captured by the apps. We made changes in the SkinCare app’s code to solve this issue. In order to deal with the operating system fragmentation, the app is designed to use only the core libraries from Android 2.3 (the first Android version that was widely used). The libraries on any later Android versions run without any problems.

The SkinCare app usage of the 13 patients participating in the field study is summarized in Table 1. The patients used the SkinCare system in varying degrees, ranging from using it only for scheduling and reminders to full-blown usage that includes pictures of skin problems and messaging to communicate with WC. It shows that patients with existing wounds tended to use SkinCare functions more than those without wounds.

**Table 1:** Patients and the SkinCheck App Feature Usage

Patient	Usage for Reminders	Messaging	Pictures	No. Body Regions	Wound locations
P1	High	High	High	3	Foot/Buttocks/Leg
P2	High	Low	Low	1	Hand
P3	Low	Low	Low	0	
P4	High	High	High	1	Back
P5	High	High	High	2	Foot/Leg
P6	Low	Low	Low	0	
P7	Low	Low	Low	0	
P8	High	High	High	1	Leg
P9	Low	Low	Low	0	
P10	Low	Low	Low	0	
P11	High	High	High	2	Foot/Arm
P12	Low	Low	Low	1	Foot
P13	High	Low	Low	2	Back/Leg

### SkinCare reminders

**Fig. 4:**
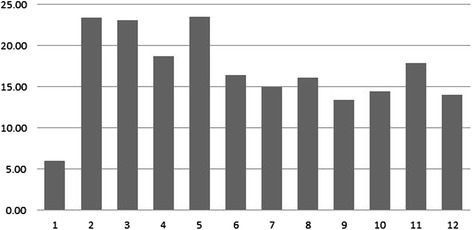
SkinCare Reminders utilization per patient per month

All patients were trained to use all apps in the iMHere system. Patients could make a decision to continue using the app or stop using it at any time during the implementation. Patients could also decide to re-start using it after stopping. Seven patients used the SkinCare app reminders for a full year, while six patients used the reminders for less than a year. The pattern of usage of the patients who consistently used the app for the entire year is illustrated in Fig 4. There was a significant increase in the first few months and then started to level off and plateau after six months.

### Utilization of SkinCare imaging

Eight of the 13 patients had wounds. All of the patients were trained on how to use imaging for skin care. All eight patients with wounds decided to use the SkinCare imaging, while patients with no wounds decided not to use the imaging. Six of the eight who used imaging had skin problems on one body part, two patients had problems on two areas of the body, and only one patient had problems on three areas of the body. Images that patients took included rashes near the knee, foot, big toe, buttocks, and lower back.

### The utilization of SkinCare messaging

The secure messaging system was developed and deployed six months into the implementation of the study. Six patients used secure messaging to communicate with WC on skin care problems and four patients became high-frequency users of the messaging (defined as sending more than 20 messages). Most of the messages were related to discussion on how to care for skin or wound problems (77 %). The rest were related to medications (10 %), skin check reminders (4 %), and arranging clinic appointments (9 %). The pattern of the messaging is a burst of messages being exchanged between a patient and WC, instead of a constant level of messaging. The spike in messaging is usually triggered by a problem requiring help from the WC. This pattern also suggests that patients used the secure messaging to discuss clinically relevant issues and did not abuse the system to merely “chat” with the WC.

### Limitations

This study demonstrates the feasibility of the SkinCAre mHealth system to support self-care of patients with skin conditions. One limitation of the study was its small sample size. The primary purpose of the study is to develop and to formatively evaluate the feasibility and usability of the mHealth system to supports self-care and clinical service delivery. For a formative evaluation, a sample size as small as five is considered sufficient to highlight usability issues of the system, and almost 100 % of the usability problems can be found using 14 participants [23, 24]. Nonetheless, the sample is small for a randomized study.

## Conclusion

Mobile health systems have the potential for supporting self-care of patients with skin conditions, either as a stand-alone system or as part of a more comprehensive system to support complex conditions. The smartphone app lends itself to supporting self-care with its reminders and scheduling that can be managed by patients. The SkinCare system presented in this paper has a unique feature of real-time bi-directional communication between a patient’s app and the clinician portal. This feature allows clinicians not only to monitor the adherence of patients to self-care tasks, but also to provide patients support on how to care for skin problems. The feasibility study conducted during a year of implementation shows that patients have been able to effectively use the SkinCare system that includes scheduling and reminders, imaging, and secure messaging. The study also shows that clinicians have been able to provide support to patients in performing self-care tasks to manage their skin conditions.

## Abbreviations

mHealth: mobile health
NIDRR: National Institute on Disability and Rehabilitation Research
SB: spina bifida
SBAWP: Spina Bifida Association of Western Pennsylvania
UTIs: urinary tract infections
WC: wellness coordinators
